# Telehealth and racial disparities in colorectal cancer screening: A pilot study of how virtual clinician characteristics influence screening intentions

**DOI:** 10.1017/cts.2022.386

**Published:** 2022-04-08

**Authors:** Eric J. Cooks, Kyle A. Duke, Jordan M. Neil, Melissa J. Vilaro, Danyell Wilson-Howard, Francois Modave, Thomas J. George, Folakemi T. Odedina, Benjamin C. Lok, Peter Carek, Eric B. Laber, Marie Davidian, Janice L. Krieger

**Affiliations:** 1 STEM Translational Communication Center, College of Journalism and Communications, University of Florida, Gainesville, FL, USA; 2 Department of Statistics, North Carolina State University, Raleigh, NC, USA; 3 Massachusetts General Hospital, Harvard University, Cambridge, MA, USA; 4 Department of Chemistry, Bethune-Cookman University, Daytona Beach, FL, USA; 5 Department of Health Outcomes and Biomedical Informatics, College of Medicine, University of Florida, Gainesville, FL, USA; 6 Division of Hematology & Oncology, Department of Medicine, College of Medicine, University of Florida, Gainesville, FL, USA; 7 Department of Pharmacotherapy and Translational Research, College of Pharmacy, University of Florida, Gainesville, FL, USA; 8 Department of Computer & Information Science & Engineering, College of Engineering, University of Florida, Gainesville, FL, USA; 9 Department of Community Health and Family Medicine, University of Florida, Gainesville, FL, USA; 10 Department of Statistical Science, Duke University, Durham, NC, USA

**Keywords:** Cancer health disparities, colorectal cancer screening, telehealth, virtual human technology, precision prevention

## Abstract

**Introduction:**

Racial disparities in colorectal cancer (CRC) can be addressed through increased adherence to screening guidelines. In real-life encounters, patients may be more willing to follow screening recommendations delivered by a race concordant clinician. The growth of telehealth to deliver care provides an opportunity to explore whether these effects translate to a virtual setting. The primary purpose of this pilot study is to explore the relationships between virtual clinician (VC) characteristics and CRC screening intentions after engagement with a telehealth intervention leveraging technology to deliver tailored CRC prevention messaging.

**Methods:**

Using a posttest-only design with three factors (VC race-matching, VC gender, intervention type), participants (*N* = 2267) were randomised to one of eight intervention treatments. Participants self-reported perceptions and behavioral intentions.

**Results:**

The benefits of matching participants with a racially similar VC trended positive but did not reach statistical significance. Specifically, race-matching positively influenced screening intentions for Black participants but not for Whites (*b* = 0.29, *p* = 0.10). Importantly, perceptions of credibility, attractiveness, and message relevance significantly influenced screening intentions and the relationship with race-matching.

**Conclusions:**

To reduce racial CRC screening disparities, investments are needed to identify patient-focused interventions to address structural barriers to screening. This study suggests that telehealth interventions that match Black patients with a Black VC can enhance perceptions of credibility and message relevance, which may then improve screening intentions. Future research is needed to examine how to increase VC credibility and attractiveness, as well as message relevance without race-matching.

## Introduction

Colorectal cancer (CRC) is the second most common cause of cancer death in the USA, and there remain substantial disparities in CRC mortality based on race [1]. Progress in reducing these mortality rates from CRC can be boosted by increasing access and adherence to recommended screening guidelines [2]. In seeking to address this issue, the United States Preventive Services Task Force (USPSTF) recently updated its recommendations for routine CRC screening to begin at 45 instead of 50 [3]. Healthy People 2030 has set a target goal of having 75% of eligible adults receive a CRC screening. Although overall CRC screening rates have improved due in part to increased investment in behavioral interventions, screening disparities account for a large portion of the racial differences seen in incidence and mortality [4]. Black patients have historically been confronted by racism, discrimination, and significant structural barriers in the US healthcare system, contributing to this racial health disparity [5,6]. While many factors contribute to these screening differences, such as financial and logistic challenges [6], one often overlooked aspect is that Black patients may be more likely to seek preventive care and follow cancer screening recommendations when delivered by a Black clinician [7,8]. This effect is likely influenced by the fact that same-race clinicians will be rated as more similar and with more positive affect [9,10]. In real-life medical encounters, race-matching between patient and clinician is difficult to align. However, new technology has created increased opportunities to address these factors.

The COVID-19 pandemic has intensified racial disparity in CRC screening and heightened the urgency to accelerate novel solutions [11]. Telehealth is increasingly being used to deliver behavioral interventions and promote uptake of home stool cancer screening modalities, including the widely used fecal immunochemical test (FIT) [12,13]. Telehealth has been defined as the exchange of medical information through electronic communication and includes a range of technologies used for various activities in health care [14,15]. In particular, web-based interactive virtual clinicians (VCs) have shown excellent potential in improving health outcomes as they allow for the delivery of anonymous interpersonal communication while also reaching large numbers of people at reduced cost [16,17]. VCs use computer-generated imagery to reproduce human visual and audio characteristics and can be easily customized to match patient demographic characteristics (e.g., race and gender). Previous research suggests that telehealth interventions providing precision CRC education via VCs can significantly impact screening intentions when patients are matched with a same-race VC [18]. However, we have yet to fully explore and disentangle this effect among Black patients, who bear the highest burden of CRC-related morbidity and mortality. To close this gap, research is needed to understand the mechanisms through which the virtual source delivering a translational communication intervention might influence cancer screening outcomes within this population.

According to the heuristic-systematic model of persuasion [19], patients will process cancer screening messages either systematically or heuristically. In systematic processing, patients closely attend to message content before forming connections between new information and pre-existing schemas. A key to engaging in this type of cognitive processing is the perception of message relevance [20]. When a cancer screening message is viewed as personally relevant, it is likely to command greater attention and systematic processing, leading to increased adherence to screening recommendations. In contrast, patients will rely on simple cues during heuristic processing to make quick judgments about the message source. For example, clinicians that are similar to patients on demographic characteristics such as race and gender may be perceived as more credible and trustworthy [21,22].

However, motivation to undergo deeper, more effortful thinking about a cancer screening message can also be influenced by source characteristics (i.e., a patient who sees a provider with similar demographic characteristics may be more motivated to process the rationale underlying why they should screen for CRC). The relationship between message source and processing suggests that demographic concordance can offer favorable heuristic judgments on message source and facilitate more scrutiny of the message argument [23]. In addition to cues that promote message processing motivation, understanding of the message content can further dictate processing type. Message format (i.e., information length, complexity, etc.) can determine the extent to which content is understood, and post-message attitudes endure. A more recent extension of the heuristic-systematic model is its application to understanding how modality interactivity can positively affect a patient’s ability to process messages [24]. As telehealth-delivered VCs offer a highly interactive message format, it may increase the patient ability to process CRC screening information.

This pilot study reports findings from a virtual translational communication intervention designed to provide patient education about CRC and facilitate FIT screening. This intervention takes a telehealth approach in order to reduce structural barriers to screening education and has the capacity for customization based on race to overcome social barriers to receiving a provider recommendation. Guided by heuristic-systematic processing, the primary purpose of this study is to test a model that assesses whether variables such as source credibility, attractiveness, and perceived message relevance are likely to influence the effect of matching the race of a VC to the race of the participant on CRC screening intentions.

## Methods

### Participants

Between November 2018 and April 2019, participants (*N* = 2267) recruited using Qualtrics Panels were randomized into the treatment conditions. After removing participants who failed to complete all dependent measures (*n* = 188) from the analysis, the final sample included 2079 participants. Eligibility criteria included: (a) US residents between the age of 50–73, (b) able to read and write in English, (c) self-identifying race as either Black or White,^1^ (d) self-reporting CRC screening status as nonadherent within screening guidelines (participants responded to three questions: colonoscopy within last 10 years, sigmoidoscopy in last 5 years, home stool test in last year; those not indicating “yes” to any of these questions were allowed to continue), and (e) providing informed consent to participate. Although CRC screening is recommended up to 75 years, we set the upper limit to 73 years to ensure eligibility for the entire duration of the study.

### Design

This pilot study examined the effects of a telehealth intervention entitled Meet ALEX (Agent Leveraging Empathy for eXams), approved by a local institutional review board. Participants who met inclusion criteria and provided informed consent were randomized to one of eight intervention treatment groups based on virtual clinician (VC) race-matching (matched, not-matched), VC gender (male, female), and intervention type (interactive, static). After completing the intervention, participants were immediately directed to the post-questionnaire. The primary purpose of this study relates to VC race-matching; other findings have been published elsewhere [18].

### Intervention

**Fig. 1.**
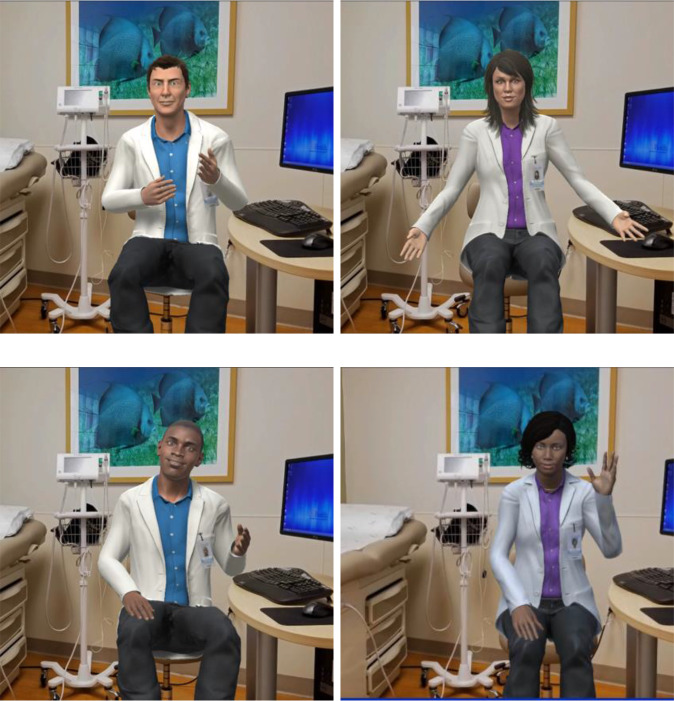
Images of the Meet ALEX (Agent Leveraging Empathy for eXams) virtual clinicians.

Applying interactive 3D technology with human voices and gestures to provide tailored CRC screening education, ALEX is a virtual clinician (VC) in a digital exam room able to engage in patient–clinician conversation (see Fig. 1). ALEX was collaboratively developed with an interdisciplinary team of communication, cancer, and computer scientists, clinicians, as well as community members [17,25].

A series of formative focus groups and think-aloud interviews with over 150 racial, ethnic, and geographically diverse community members within the research team’s largely rural catchment area informed the user-centered design of Meet ALEX. Phase 1 collected information about the preferences and needs of potential users before prototype development. In Phase 2 users tested the prototype and commented on aspects of the VC and the messaging content. After further refinement Phase 3 included additional feedback on the appearance of the VCs, which led to final modification. Efforts were made to reduce the health literacy required to engage with ALEX (i.e., providing visuals of how to collect stool samples rather than words). The interactive ALEX provides information verbally with closed captioning, asks closed-ended questions, and uses nonverbal behaviors; patients receive personalized prevention messaging based on their responses (see supplement for link). The static ALEX provides identical content in text format along with still photos. The intervention addresses various modalities of screening (colonoscopy, Cologuard, FOBT, FIT) with a particular emphasis on the ease and affordability of FIT.

### Measures

#### Race-matching

Race-matching with the VC was assessed categorically; participants randomized to interact with a same-race VC were classified as “matched,” while those engaging with a racially dissimilar VC were labeled as “not matched.”

#### VC credibility

Perceived source credibility [26] was measured using five 5-point semantic differential items with labels (unintelligent-intelligent; untrustworthy-trustworthy; inexpert-expert; uninformed-informed; incompetent-competent). The item read, “ALEX was your virtual healthcare assistant who provided you the health information during your virtual appointment. ALEX is….” All five items correlated with each other (*r* >0.61), and an exploratory factor analysis suggests one underlying factor (Eigenvalue 3.78), with factor loadings of between 0.79 and 0.90. The median credibility score across these five items indicated perceived source credibility (α = 0.92, Mdn = 4.59, SD = 0.67).

#### VC attractiveness

Using a 5-point semantic differential item with the same prompt as the credibility measure, ratings of VC attractiveness were assessed with the label (unattractive-attractive) (M = 3.73, SD = 0.96) [27].

#### Perceived message relevance

Participant perceptions of message relevance were also measured with a single item [28]. On a 5-point Likert scale (“strongly disagree” to “strongly agree”), participants were asked how much they agree with the following statement, “The information received was customized to me.” (M = 3.48, SD = 1.19).

#### CRC screening intentions

The ALEX intervention provides education on CRC screening modalities, including FIT, and demonstrates correct stool collection. Intention to screen for CRC was measured using a single item (i.e., “I will talk to my clinician about colon cancer screening with FIT”) on a five-point scale with response options ranging from “strongly disagree” to “strongly agree” (M = 3.73, SD = 1.27).

#### Covariates

Participant age and sex were included as controls in the statistical models. Age was measured continuously. Sex was determined by asking participants if they more closely identify with the term “Man” or “Woman.”

### Statistical Analysis

Statistical analyses were two-sided and conducted using R 4.0.1 (R Core Team, 2018). Logistic regression models were used with the *clm* function in the *ordinal* package. Models contained up to two-way interactions between the experimental factors of interest (VC race-matching and intervention type) and participant race. Effects of participant age and sex were included in all models as a control on potential confounding factors. Preliminary analyses showed no main effects of VC gender, so it was not included in subsequent models.

We used these models to explore whether the perceived credibility and attractiveness of the VC, along with the perceived message relevance of the patient education provided during the intervention, mediate the effect of matching the race of the VC to the race of the participant on CRC screening intentions. Mediation analyses follow a three-step process: (1) the relationship between the independent variable and the dependent variable is identified, followed by examining (2) the relationship between the independent variable and the potential mediator, and finally (3) the relationship between the independent variable and the dependent variable in the presence of the mediator is explored. We considered the interaction models that included the participant race and VC race-matching interaction as the independent variable, screening intentions as the dependent variable, and perceived credibility, attractiveness, and message relevance as mediators. Given the three potential mediators, three separate mediation analyses were conducted. We interpreted the presence of mediation effects by examining changes in effect sizes of the interaction between the initial model and the subsequent model with the potential mediator as an additional main effect.

## Results

### Participant Characteristics

Participant characteristics are presented in Table 1. Participants were primarily female (69.9%) with an average age of 58.7 (SD = 6.2). Within this sample, 37.1% (*n* = 840) of participants self-reported as Black, and 62.9% (*n* = 1427) as White. Over 60% of participants had at least some post-secondary education and participant income level was well distributed. Table 2 provides percentages of participant responses at each level on the 5-point scale for VC credibility, VC attractiveness, message relevance, and CRC screening intention. Table 3 offers a similar breakdown based on the interaction levels of participant race and VC race-matching.

**Table 1.** Sociodemographic characteristics of participants (*N* = 2267)

Characteristic	*n* (%)
Age, mean (SD)	58.7 (6.2)
Gender, No. (%)	
Man	908 (40.1%)
Woman	1359 (59.9%)
Race, No. (%)	
Black	840 (37.1%)
White	1427 (62.9%)
Education	
College graduate	475 (22.8%)
Technical, trade or vocational school AFTER high school	559 (26.9%)
High school incomplete (completed grades 1–8)	41 (2.0%)
Post-graduate training/professional school after college (M.A., Ph.D., JD, or MD)	195 (9.4%)
Some college	809 (38.9%)
Income	
Less than $10,000	142 (6.8%)
$10–$20,000	298 (14.3%)
$20–$30,000	338 (16.3%)
$30–$40,000	246 (11.8%)
$40–$50,000	198 (9.5%)
$50–$75,000	336 (16.2%)
$75–$100,000	204 (9.8%)
$100,000 or more	158 (7.6%)

**Table 2.** Proportion of participant responses across items, overall and by race

Study variable	Overall % (*n*)	Participant race % (*n)*	
		Black	White
*VC credibility*			
1	0.4 (9)	0.2 (1)	0.06 (8)
2	0.3 (6)	0.3 (2)	0.03 (4)
3	6.7 (139)	4.4 (28)	7.8 (111)
4	25.4 (523)	17.0 (109)	29.1 (414)
5	67.2 (1386)	78.2 (502)	62.2 (884)
*VC attractiveness*			
1	1.9 (40)	1.5 (10)	2.1 (30)
2	2.9 (60)	2.0 (13)	3.3 (47)
3	43.0 (889)	34.1 (220)	47.0 (669)
4	25.0 (516)	24.1 (156)	25.3 (360)
5	27.2 (563)	38.2 (247)	22.2 (316)
*Message relevance*			
1	7.5 (156)	6.7 (44)	7.8 (112)
2	12.1 (251)	9.8 (64)	13.1 (187)
3	29.1 (606)	24.2 (158)	31.4 (448)
4	27.5 (571)	26.2 (171)	28.0 (400)
5	23.8 (495)	33.0 (215)	19.6 (280)
*CRC screening intention*			
1	9.0 (188)	5.4 (35)	10.7 (153)
2	7.6 (157)	5.4 (35)	8.5 (122)
3	21.2 (441)	16.4 (107)	23.4 (334)
4	25.5 (531)	22.4 (146)	27.0 (385)
5	36.7 (762)	50.5 (329)	30.3 (433)

Abbreviation: VC, virtual clinician. CRC, colorectal cancer.

**Table 3.** Proportion of participant responses across items, overall and by participant race/virtual clinician (VC) race-matching

Study variable	Overall % (*n*)	Participant race/race-matching % (*n)*			
		Black/matched	Black/mis-matched	White/matched	White/mis-matched
*VC credibility*					
1	0.4 (9)	0.0 (0)	0.3 (1)	1.3 (7)	0.1 (1)
2	0.3 (6)	0.2 (1)	0.3(1)	0.6 (3)	0.1 (1)
3	6.7 (139)	3.8 (17)	2.8 (11)	9.1 (43)	7.6 (68)
4	25.4 (523)	10.8 (48)	15.4 (61)	32.9 (175)	26.7 (239)
5	67.2 (1386)	59.3 (264)	60.3 (238)	57.0 (303)	64.9 (581)
*VC attractiveness*					
1	1.9 (40)	0.9 (4)	1.5 (6)	3.4 (18)	1.3 (12)
2	2.9 (60)	1.6 (7)	1.5 (6)	3.4 (18)	3.2 (29)
3	43.0 (889)	22.5 (100)	30.4 (120)	52.3 (278)	43.7 (391)
4	25.0 (516)	17.3 (77)	20.0 (79)	25.6 (136)	25.0 (224)
5	27.2 (563)	32.6 (145)	25.8 (102)	15.2 (81)	26.3 (235)
*Message relevance*					
1	7.5 (156)	6.5 (29)	3.8 (15)	8.3 (44)	7.6 (68)
2	12.1 (251)	6.1 (27)	9.4 (37)	14.5 (77)	12.3 (110
3	29.1 (606)	15.1 (67)	23.0 (91)	34.2 (182)	29.7 (266)
4	27.5 (571)	19.6 (87)	21.3 (84)	24.2 (129)	30.3 (271)
5	23.8 (495)	28.3 (126)	22.5 (89)	18.8 (100)	20.1 (180)
*CRC Screening Intention*					
1	9.0 (188)	3.4 (16)	5.1 (20)	11.7 (62)	10.2 (91)
2	7.6 (157)	3.6 (16)	4.8 (19)	8.8 (47)	8.4 (75)
3	21.2 (441)	13.7 (61)	11.6 (46)	24.1 (128)	23.0 (206)
4	25.5 (531)	14.8 (66)	20.3 (80)	28.4 (151)	26.1 (234)
5	36.7 (762)	40.0 (178)	38.2 (151)	27.1 (144)	32.3 (289)

Abbreviation: VC, virtual clinician. CRC, colorectal cancer.

### Effects for CRC Screening Intention

**Fig. 2.**
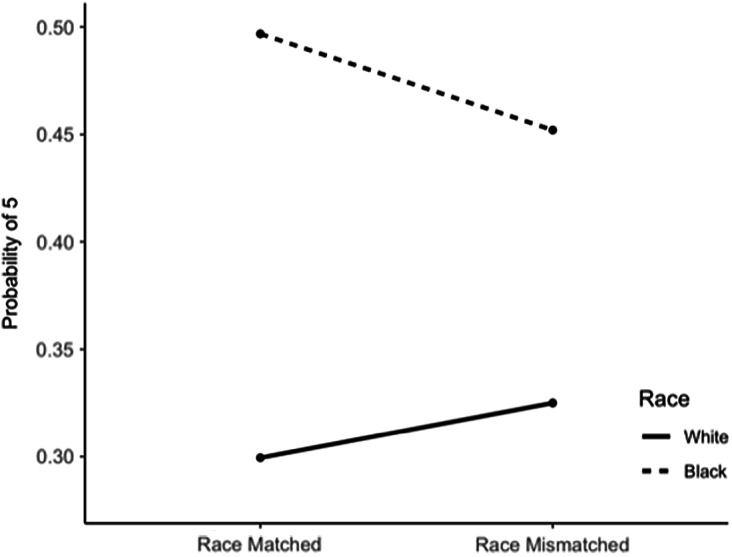
Interaction effect of participant race and virtual clinician (VC) race-matching on colorectal cancer (CRC) screening intention.

The analysis revealed that after engaging with the intervention, Black participants overall reported significantly higher screening intentions compared to Whites (*b* = 0.53, *p* < 0.001, 95% CI = 0.23–0.84) (see Table 4). The statistical benefits of matching virtual clinician (VC) race to participant race on screening intentions were mixed. Black participants reported higher screening intentions after visiting with a Black VC (*b* = 0.12, *p* = 0.46, 95% CI = −0.21 to 0.45) as compared to a White VC. For White participants, screening intentions were lower after a virtual appointment with a White VC relative to a Black VC (*b* = −0.17, *p* = 0.18, 95% CI = −0.42 to 0.08). In sum, matching the race of the VC to the race of the participant trended a favorable but statistically nonsignificant influence on screening intentions for Black participants, but not for Whites (b = 0.29, *p* = 0.10, 95% CI = 0.05–0.64) (Fig. 2).

**Table 4.** Regression of participant race, race-matching, and intervention type on virtual clinician (VC) credibility, VC attractiveness, message relevance, and colorectal cancer (CRC) screening intention

Predictor variable	VC credibility			VC attractiveness			Message relevance			CRC screening intention		
	*B (SE)*	P*	95% *CI*	*B (SE)*	P*	95% *CI*	*B (SE)*	P*	95% *CI*	*B (SE)*	P*	95% *CI*
*Covariates*												
Age	0.002 (0.008)	0.8	(−0.01, 0.02)	0.01 (0.01)	0.41	(−0.01, 0.02)	−0.03 (0.01)	<0.001	(−0.04, −0.02)	−0.02 (0.01)	<0.001	(−0.04, −0.01)
Sex (women)	0.34 (0.10)	<0.001	(0.15, 0.53)	0.58 (0.09)	<0.001	(0.41, 0.75)	0.25 (0.08)	0.002	(0.09, 0.41)	0.22 (0.08)	0.009	(0.05, 0.38)
*Main effects*												
Black vs. white participants (ref)	0.38 (0.18)	0.04	(0.03, 0.75)	0.11 (0.16)	0.49	(−0.20, 0.41)	0.24 (0.15)	0.11	(−0.06, 0.54)	0.53 (0.15)	<0.001	(0.23, 0.84)
Matched vs. not-matched (ref)	−0.25 (0.14)	0.09	(−0.53, 0.04)	−0.34 (0.13)	0.01	(−0.61, −0.08)	−0.20 (0.13)	0.12	(−0.45, 0.05)	−0.17 (0.13)	0.18	(−0.42, 0.08)
Interactive VC vs. static VC (ref)	0.45 (0.13)	0.001	(0.19, 0.72)	0.41 (0.12)	<0.001	(0.18, 0.64)	0.26 (0.11)	0.02	(0.04, 0.48)	0.14 (0.11)	0.22	(−0.08, 0.36)
*Interactions*												
VC type × race-matching	−0.14 (0.19)	0.47	(−0.51, 0.24)	−0.19 (0.17)	0.26	(−0.52, 0.14)	0.12 (0.16)	0.45	(−0.20, 0.44)	0.11 (0.16)	0.51	(−0.21, 0.43)
VC type × participant race	0.16 (0.22)	0.48	(−0.28, 0.60)	0.07 (0.18)	0.69	(−0.28, 0.42)	−0.16 (0.17)	0.35	(−0.50, 0.18)	0.02 (0.18)	0.92	(−0.32, 0.36)
Race-matching × participant race	0.56 (0.22)	0.01	(0.12, 1.00)	0.93 (0.18)	<0.001	(0.58, 1.29)	0.47 (0.18)	0.01	(0.13, 0.82)	0.29 (0.18)	0.1	(−0.05, 0.64)

Abbreviation: VC, virtual clinician. CRC, colorectal cancer.

* Regression analyses. All statistical tests were two-sided. *B* = unstandardized beta-coefficient; *CI* = confidence interval.

### Effects for VC Credibility, VC Attractiveness, and Message Relevance

#### VC credibility

**Fig. 3.**
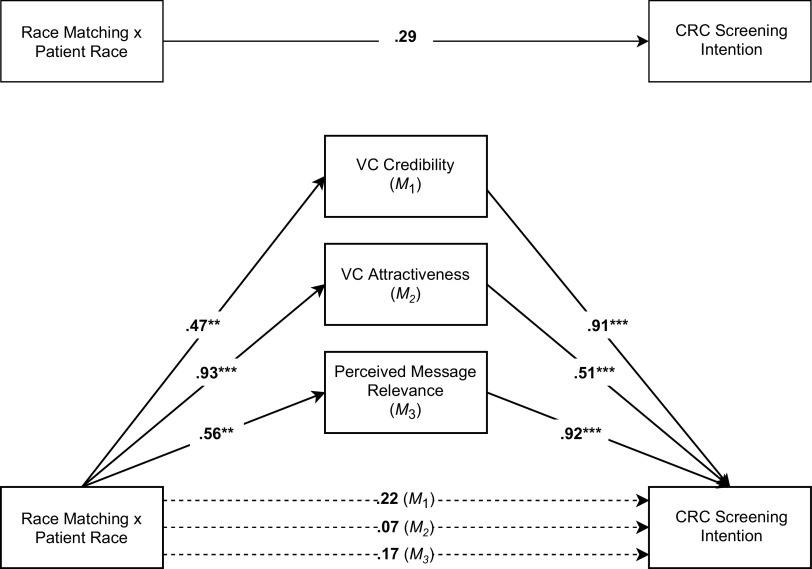
A conceptual model of parallel mediation depicting the indirect effects of race-matching x participant race through virtual clinician (VC) credibility, VC attractiveness, and perceived message relevance on participant colorectal cancer (CRC) screening intention. *Note.* Three direct effects of race-matching × participant race on intent are presented, each controlling for the mediator in the regression model (*M*1, *M*2, *M*3); Solid lines represent statistically significant relationships; *P < 0.05; **P < 0.01; **P < 0.001.

As pathways in the larger model (Fig. 3), Table 4 also presents the effects of intervention components on perceived VC credibility and attractiveness, as well as message relevance. Among all participants, those who engaged with the interactive VC found it to be more credible compared to the static condition (*b* = 0.45, *p* = 0.001, 95% CI = 0.19–0.72). Regardless of VC race or gender, Black participants evaluated the VC as significantly more credible than did Whites (*b* = 0.38, *p* = 0.04, 95% CI = 0.03–0.75). Further, matching the race of the VC to the race of the participant contributed to higher ratings of credibility for Black participants than it did for Whites (*b* = 0.56, *p* = 0.01, 95% CI = 0.12–1.00).

#### VC attractiveness

Regardless of race, participants who engaged with the interactive VC reported significantly higher attractiveness (*b* = 0.41, *p* < 0.001, 95% CI = 0.18–0.64) compared to the static VC. Black participants found the Black VC to be significantly more attractive than the White VC (*b* = 0.59, *p* < 0.001, 95% CI = 0.26–0.92). In addition, while participants overall reported higher attractiveness when they were not race-matched with a VC (*b* = −0.34, *p* = 0.01, 95% CI = −0.61 to −0.08), this effect was significantly influenced by participant race; both Black and White participants reported higher attractiveness for the Black VC (*b* = 0.93, *p* < 0.001, 95% CI = 0.58–1.29).

#### Message relevance

Regarding message relevance, effects continued to be seen based on intervention type. After visiting with the interactive VC, participants evaluated the CRC prevention education as significantly more relevant (*b* = 0.26, *p* = 0.02, 95% CI = 0.04–0.48). The advantage of matching VC race to participant on perceived message relevance was apparent; race-matching positively influenced message relevance for Black participants (*b* = 0.47, *p* = 0.01, 95% CI = 0.13–0.82) compared to Whites.

### Mediation Effects

To test the potential that race is a heuristic cue for other variables essential to effective health communication, we examined the relationships among the proposed mediators and the primary outcome. The data revealed that the perceived attractiveness (*b* = 0.51, *p* < 0.001, 95% CI = 0.42–0.60) and credibility (*b* = 0.92, *p* < 0.001, 95% CI = 0.80–1.05) of the VC, as well as the perceived message relevance (*b* = 0.91 *p* < 0.001, 95% CI = 0.83–0.99) all positively predict CRC screening intentions (Table 5 and Fig. 3).

**Table 5.** Path coefficients from the parallel mediation model illustrated in Fig. 3

Predictor variable	VC credibility			VC attractiveness			Message relevance			CRC screening intention		
	*B (SE)*	P*	95% *CI*	*B (SE)*	P*	*95% CI*	*B (SE)*	P*	95% *CI*	*B (SE)*	P*	95% *CI*
*Interaction*												
Race-matching × participant race	0.56 (0.22)	0.01	(0.12, 1.00)	0.93 (0.18)	<0.001	(0.58, 1.29)	0.47 (0.18)	0.01	(0.13, 0.82)	0.29 (0.18)	0.10	(−0.05, 0.64)
*Mediator effects*												
Message relevance^M1^	–	–	–	–	–	–	–	–	–	0.91 (0.04)	<0.001	(0.83, 0.99)
VC attractiveness^M2^	–	–	–	–	–	–	–	–	–	0.51 (0.05)	<0.001	(0.42, 0.60)
VC source credibility^M3^	–	–	–	–	–	–	–	–	–	0.92 (0.06)	<0.001	(0.80, 1.05)
*Adjusted interaction effects*												
Race-matching × participant race^M1^	–	–	–	–	–	–	–	–	–	0.22 (0.19)	0.25	(−0.15, 0.58)
Race-matching × participant race^M2^	–	–	–	–	–	–	–	–	–	0.07 (0.18)	0.70	(−0.28, 0.43)
Race-matching × participant race^M3^	–	–	–	–	–	–	–	–	–	0.17 (0.18)	0.34	(−0.18, 0.53)

Abbreviation: VC, virtual clinician. CRC, colorectal cancer.

*Note:* Em-dash represents a relationship that was not regressed upon as part of the model.

* Parallel mediation conducted using the *ordinal* package in R 4.0.1. All statistical tests were two-sided. *B* = unstandardized beta-coefficient; *CI* = confidence interval.

To show mediation, the effect of matching VC race to participant race on screening intentions should be reduced when accounting for the proposed mediators. Taking attractiveness ratings into account, the strength of this effect was reduced (*b* = 0.29, *p* = 0.10 to *b* = 0.07, *p* = 0.70). Similarly, in the presence of the main effect of credibility, this effect was diminished in strength (*b* = 0.29, *p* = 0.10 to *b* = 0.17, *p* = 0.34). This interaction effect also decreased in strength after accounting for perceived message relevance (*b* = 0.29, *p* = 0.10 to *b* = 0.22, *p* = 0.25).

## Discussion

The primary goal of this study was to explore whether VC characteristics (i.e., perceived credibility and attractiveness) and the perceived relevance of CRC prevention messaging might shape the impact of matching VC race to participant race on CRC screening intentions. Contrary to the hypothesis, VC race-matching was not a statistically significant predictor of screening intentions. However, Black participants overall reported higher screening intentions after the intervention, and there was a trend indicating that this effect was amplified when receiving a Black VC. Importantly, there were three important mediators of the relationship between race-matching and CRC intentions: source credibility, attractiveness, and message relevance. In other words, race matching positively influenced perceptions of VC characteristics and message relevance, which were associated with CRC screening intention. The findings of this pilot study suggest that being race-matched with a VC is an important but not sufficient cue for CRC screening intention among Black patients and offer critical insight for the development of precision telehealth interventions to address racial disparities in CRC screening.

Evaluating these results through the lens of the heuristic-systematic model, this study provides compelling evidence that heuristic cues can help facilitate systematic processing of cancer prevention messaging. Specifically, VC race appears to act as a heuristic cue leading participants to perceive the CRC prevention messaging components delivered by ALEX as personally relevant. It is fundamental across healthcare contexts to improve awareness of the potential influence of visual cues on how patient education is processed. Telehealth interventions such as Meet ALEX are uniquely situated to adapt intervention components to the needs of patients, such as racially matched providers. We expect the greatest return on this investment to reflect patients more accurately will be for underrepresented populations, who face more challenges to screening and are often diagnosed with CRC at a later stage. Additional research is needed to explore how other racial/ethnic minority groups facing disparities in CRC screening (e.g., Hispanics, Indian Americans) respond to the intervention.

Another noteworthy finding was that the interactive VC was rated more favorably than the static version across conditions, emphasizing the importance of interactivity in cancer screening interventions. Previous research has explored interactivity in online cancer information and found that patients prefer two-way communication exchange and active control over their experience [29]. The ALEX intervention addresses these needs by utilizing interactive VCs to engage in reciprocal communication while providing patients with the opportunity to self-guide their experiences.

Engaging with a Black VC was a necessary cue for attractiveness among Black participants; however, attractiveness was also a significant predictor of screening intention among all participants. From these data, we do not know what influenced these high attractiveness ratings among White participants, although potential explanations include voice features and presumed age of the VC; these intervention factors were developed and refined by including patient perspective during the iterative, user-centered design stage [25]. These findings suggest that scientists, clinicians, and stakeholders should devote adequate resources to enhancing the appearance of virtual clinicians delivering CRC prevention messaging. Prior research suggests that the appearance of these virtual agents may have a positive influence on message persuasiveness and patient motivation to engage in preventive behavior [30–32].

Furthermore, for Black patients confronted with significant racial disparities in health and structural barriers to CRC screening, the perceived credibility of a clinician is likely to be extremely consequential; these patient groups may look for specific cues to make determinations on credibility and trust, including racial similarity and attractiveness [27]. The relationship between these cues and CRC screening intention is crucial as telehealth interventions such as Meet ALEX have the capacity to reduce screening barriers for Black patients through remote delivery and increased knowledge of affordable at-home screening options such as the FIT. These findings also highlight the magnitude of efforts to diversify the healthcare workforce. For groups that have not endured these historical inequalities, there may be less uncertainty surrounding clinician credibility, thus reducing the impact of this factor within these populations.

The results of this pilot study extend previous work [18] by suggesting that patient-focused telehealth interventions that match Black patients with a Black VC have significant effects on perceptions of source characteristics and message relevance. These perceptions may be a key factor in CRC screening intentions. In real-life medical encounters, race-matched interactions between patients and clinicians can increase perceived credibility and strengthen adherence to cancer screening recommendations [33–35]. At the same time, in this virtual setting, VC attractiveness is likely to influence both persuasiveness and patient motivation [36]. The model developed and tested in this study informs predictions for patient response to VC characteristics. Statistically significant effects were identified for race-matching on the mediators (credibility, attractiveness, and perceived message relevance). However, while positive trends were uncovered, this significance did not extend to screening intention. To understand the effects of this intervention more thoroughly on screening behaviors and intentions among Black patients, additional research is needed as part of a controlled clinical trial involving active and engaged patients with documented access to primary care. Future research should also explore approaches to increasing intervention capacity for offering credible and attractive VCs that deliver relevant CRC prevention messaging without race-matching. Further, the effects of other features made available through virtual humans should also be explored, such as the tailoring of voice, intonation, and ethnic similarity.

It is worth noting that this study was conducted before the COVID-19 pandemic, which saw rapid adoption of telehealth [37], suggesting that barriers to healthcare access, including cost and transportation, which are mitigated by telehealth, are more likely to have affected this sample. Taken together, these findings hold practical implications for the development of telehealth interventions to address structural barriers, particularly among racial minorities who are less likely to get screened and are more likely to be diagnosed at a late stage. Integration of VCs into the clinical workflow can improve communication by providing patients with an opportunity to select how health promotion information is delivered. For providers, VCs can serve as a reinforcement after a patient care visit or as an introduction to health promotion topics, in this case CRC screening. Tools such as the ALEX intervention can be more fully integrated across health systems through patient portals, where a demographically matched VC can provide predeveloped information (e.g., upcoming appointments, prescriptions) or more nuanced education (e.g., cancer screening) that can then be championed by providers.

### Limitations

This exploratory study is not without limitations. Participants were recruited through Qualtrics, which utilizes national panels. These individuals may be more familiar with online platforms; however, online panels such as Qualtrics can effectively recruit diverse samples for cancer research [38]. The study measured screening intentions rather than behavioral outcomes. Participants were not tracked to determine whether they engaged in discussions with a clinician or took any recommended screening actions. Yet, there is strong evidence of an association between CRC screening intention and behavior [39,40]. Data also were not collected on whether participants had ever been screened; this is an important future direction for research.

Furthermore, although a large number of Blacks were included, Whites were overrepresented in this sample as is typical in nationally representative studies. It is imperative to focus on strategies that are important to Black patients, especially those that might be not observed in other groups. While this sample may not fully reflect the demographic profile of the typical nonadherent patient, the participants in this study are outside of guidelines, and the general purpose of this intervention is to get people up to date with screening and to help them overcome challenges in doing that. This pilot is a test of efficacy before moving into a clinical population. Given recent changes in CRC screening guidelines to include patients 45–49 years of age, it is important to examine best practices for communicating these new guidelines to patients who might have never heard them before. Additional research is also needed to explore perceptions of Black patients in this new age group. Previous positive or negative interactions with the healthcare system or pre-existing relationships with human clinicians were not analyzed, potentially introducing additional biases in the study findings. In addition, although race-matching with a VC displayed a pattern of higher intentions to discuss FIT among Black participants, we do not know how the race of one’s human clinician influences this effect. In the clinic, this could be bypassed by having the VC place an order for FIT through a patient portal, removing the need for face-to-face interaction.

## Conclusions

As the ability to deliver care through telehealth and interactive VCs continues to emerge as a means of overcoming patient barriers, the scope of patient–clinician communication research must be broadened to disentangle the effects of these digital technologies on patient outcomes. Although efforts are underway to diversify the real-life healthcare workforce [41], there is still a shortage of minority healthcare clinicians. VCs provide an easily accessible tool to remind and motivate patients to take fast action to stay in guidelines and can be easily tailored to patient preferences and can help get important prevention information to patients and their families in a culturally sensitive manner. Taken together, findings from this study suggest that telehealth interventions developed to reduce racial disparities in CRC screening that match Black patients with an interactive, Black VC can maximize perceptions of credibility and message relevance, which may then positively influence patient screening intentions.

## Footnotes

1 The current iteration of the Meet ALEX intervention can be customized on race only for Black and White patients and to test the effects of race-matching only Black and White participants were recruited.

## Supplementary material

For supplementary material accompanying this paper visit https://doi.org/10.1017/cts.2022.386. click here to view supplementary material
