# Supplementary material for: Telehealth and racial disparities in colorectal cancer screening: A pilot study of how virtual clinician characteristics influence screening intentions
Source: J Clin Transl Sci. 2022 Apr 8;6(1):e48. doi: 10.1017/cts.2022.386 (PMC9108377; doi:10.1017/cts.2022.386)
Supplement: Supplementary file 1 [file S2059866122003867sup001.docx]

**Technical Supplement**

The 2267 participants in this study included members of a nationally representative opt-in panel maintained by Qualtrics of individuals who have agreed to participate in research studies. Panel members were invited to through an emailed link and completed eligibility screening questions. Through the web-based crowdsourcing survey process provided by Qualtrics panels, 24,732 participants began the survey, and more than 22,000 were screened out. The primary outcome of the study was intention to talk to a provider about CRC screening with FIT, which was measured using a 5-point Likert scale. The initial goal of the statistical analysis was to identify a relationship between participant race and two, binary experimental factors: VC race-matching and VC type. Once some evidence of a relationship between VC race-matching and patient race was identified, we sought to identify whether VC credibility, VC attractiveness, and perceived message relevance were mediators of this effect. VC credibility, VC attractiveness, and perceived message relevance each were measured using a 5-point Likert scale. All of the constructed models in this statistical analysis were ordinal regression models with a logit link function.

We refer to the ordinal regression model with CRC screening intentions as the dependent variable and patient race, VC race-matching, and VC type as predictors as the baseline model. The baseline included all two-way interactions between patient race, VC race-matching, and VC type. The model also included patient age and patient gender as main effects. Patient age and gender were identified as confounding variables, as there was a relationship between patient race and both age and gender within the sample. Through an exploratory analysis, patient race and gender were found to be correlated with intentions to talk to a doctor about CRC screening with FIT. After constructing the baseline model, we separately regressed the three potential mediators against the same predictors contained in the baseline model: all two-way interactions between patient race, VC racing-matching, VC type with age and gender included as main effects. We refer to the three intermediary models corresponding to VC credibility, VC attractiveness, and perceived message relevance as M1, M2, and M3, respectively. We identified a statistically significant effect of the interaction between patient race and VC race-matching in each of the three intermediary models. Finally, to identify full or partial mediation, we included each of the mediators separately as main effects in the baseline model, for a total of seven ordinal regression models. We refer to the three intentions models which include the main effects of VC credibility, VC attractiveness, and perceived message relevance as Int1, Int2, and Int3, respectively. For simplicity of both presentation and interpretation, in the final mediation models, we included VC credibility, VC attractiveness, and perceived message relevance as continuous variables instead of categorical. The results of the analysis were invariant to how these three variables were coded as predictors.

Two-sided Z-tests were used to test whether regression coefficients had statistically significant effects on the dependent variables. Figure 1 shows a QQ plot, supporting the distributional assumptions on the residuals. Figure 2 includes a residual plot demonstrating a logistic distribution.

Of the 2267 participants, 2079 were used in the baseline model regression analysis. The excluded patients did not complete the survey and did not report any intentions to talk to a doctor about CRC screening. The amount of missing data excluded from the analysis varied by model, as the seven regression models included different dependent variables and predictors. More details on the amount of missing data can be found in table 1.

Table 2 contains regression summaries of the models Int1, Int2, and Int3 for parameters which were omitted from the main paper.

The Meet ALEX VC can be found through the following link: <http://www.virtualpeoplefactory.com/Classic/Interaction/Prototype_CRC_PilotB/99?username=ce-vh-bf>.


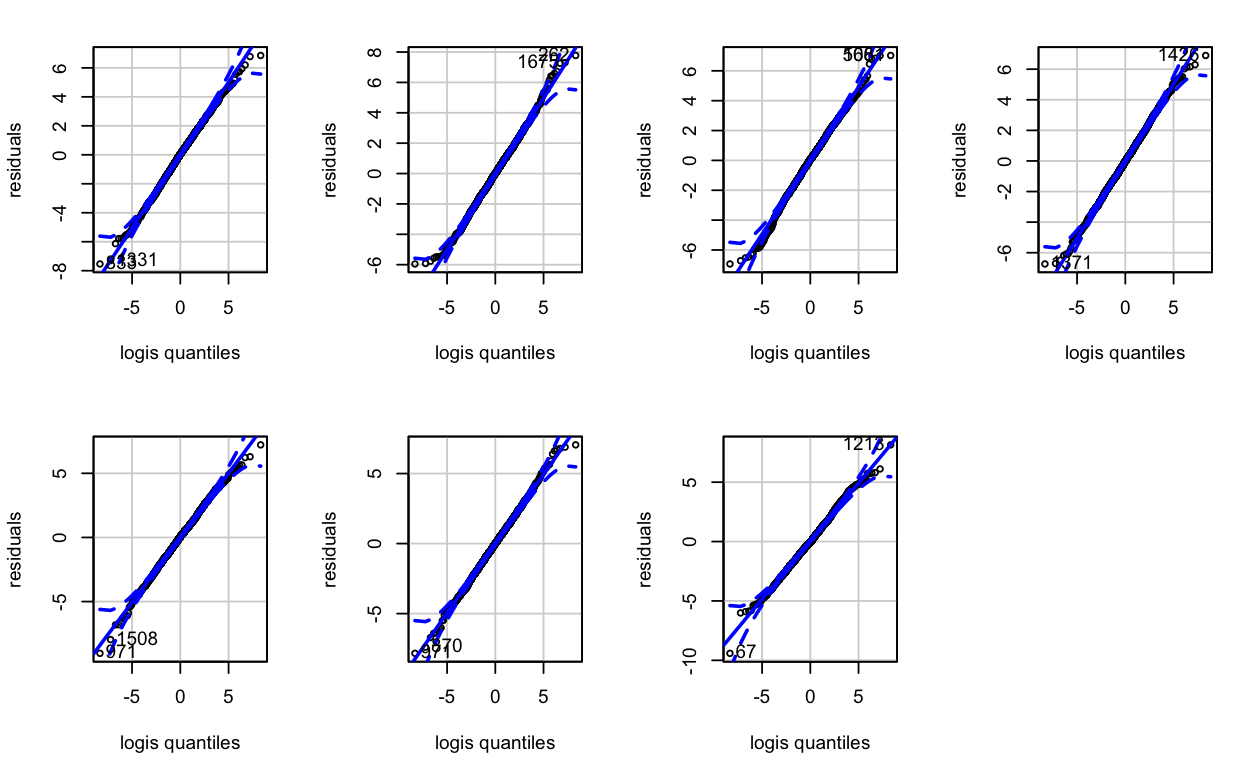


Figure 1: QQ plots indicating logistic distribution of residuals.


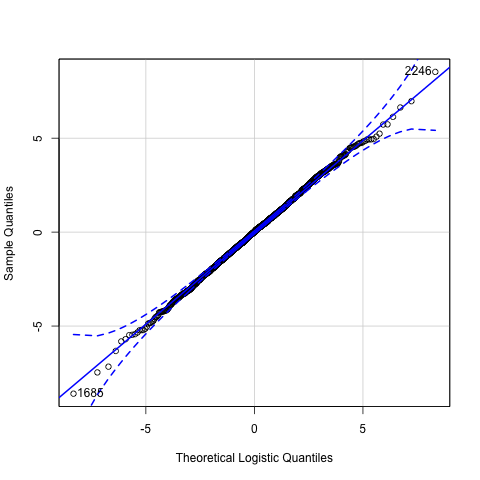


Figure 2: Residual plot on CRC screening intention for logistic analysis.

| Table 1. Summary of the number of patients used to fit each regression model. The diagonal entries contain the number patients used to fit each of the seven models. The off-diagonal entries contain the number of patients which were used to fit both corresponding models. | | | | | | | |
| --- | --- | --- | --- | --- | --- | --- | --- |
|  | Baseline | M1 | M2 | M3 | Int1 | Int2 | Int3 |
| Patients Analyzed | 2079 | 2063 | 2068 | 2079 | 2061 | 2066 | 2078 |

| Table 2. Regression summary for Int1, Int2, and Int3. Main effects of the mediators are omitted and can be found in the main document. | | | | | | | | | |
| --- | --- | --- | --- | --- | --- | --- | --- | --- | --- |
|  | **Perceived Message Relevance** | | | **VC Attractiveness** | | | **VC Source Credibility** | | |
| **Predictor Variable** | ***B (SE)*** | ***P*** | **95% *CI*** | ***B (SE)*** | ***P*** | **95% *CI*** | ***B (SE)*** | ***P*** | **95% *CI*** |
| *Covariates* |  |  |  |  |  |  |  |  |  |
| Age | -0.008 (0.007) | 0.216 | (-0.02, 0.01) | -0.03 (0.01) | <0.001 | (-0.04,  -0.01) | -0.02 (0.01) | <0.001 | (-0.04,  -0.01) |
| Sex (Women) | 0.13 (0.09) | 0.137 | (-0.05, 0.30) | 0.09 (0.09) | 0.267 | (-0.07, 0.26) | 0.13 (0.08) | 0.115 | (0.03, 0.30) |
| *Main Effects* |  |  |  |  |  |  |  |  |  |
| Black vs. White Participants (ref) | 0.49 (0.16) | 0.002 | (0.17, 0.80) | 0.54 (0.16) | 0.001 | (0.24, 0.85) | 0.50 (0.16) | 0.002 | (0.19, 0.81) |
| Concordant vs. Discordant (ref) | -0.08 (0.13) | 0.541 | (-0.34, 0.18) | -0.06, (0.13) | 0.644 | (-0.31, 0.19) | -0.07 (0.13) | 0.594 | (-0.32, 0.19) |
| Interactive VC (ref) vs. Static VC | 0.04 (0.12) | 0.705 | (-0.18, 0.27) | 0.05 (0.11) | 0.690 | (-0.18, 0.27) | 0.05 (0.11) | 0.689 | (-0.18, 0.27) |
| *Interactions* |  |  |  |  |  |  |  |  |  |
| VC Type x Race-Matching | 0.03 (0.17) | 0.861 | (-0.30, 0.36) | 0.12 (0.16) | 0.449 | (-0.20, 0.45) | 0.09 (0.17) | 0.571 | (-0.23, 0.52) |
| VC Type x Participant Race | 0.07 (0.19) | 0.373 | (-0.29, 0.43) | 0.01 (0.18) | 0.974 | (-0.35, 0.36) | -0.03 (0.18) | 0.859 | (-0.38, 0.32) |
| Race-Matching x Participant Race | 0.22 (0.19) | 0.247 | (-0.15, 0.58) | 0.07 (0.18) | 0.704 | (-0.29, 0.43) | 0.17 (0.18) | 0.338 | (-0.18, 0.53) |
